# Cytolytic Perforin as an Adjuvant to Enhance the Immunogenicity of DNA Vaccines

**DOI:** 10.3390/vaccines7020038

**Published:** 2019-04-30

**Authors:** Ashish C. Shrestha, Danushka K. Wijesundara, Makutiro G. Masavuli, Zelalem A. Mekonnen, Eric J. Gowans, Branka Grubor-Bauk

**Affiliations:** Virology Laboratory, Discipline of Surgery, Basil Hetzel Institute for Translational Health Research and University of Adelaide, Adelaide 5011, Australia; danushka.wijesundara@adelaide.edu.au (D.K.W.); makutiro.masavuli@adelaide.edu.au (M.G.M.); zelalem.mekonnen@adelaide.edu.au (Z.A.M.); eric.gowans@adelaide.edu.au (E.J.G.)

**Keywords:** DNA vaccine, adjuvants, vaccine delivery, plasmid, cytolytic, perforin, bicistronic, HCV, HIV

## Abstract

DNA vaccines present one of the most cost-effective platforms to develop global vaccines, which have been tested for nearly three decades in preclinical and clinical settings with some success in the clinic. However, one of the major challenges for the development of DNA vaccines is their poor immunogenicity in humans, which has led to refinements in DNA delivery, dosage in prime/boost regimens and the inclusion of adjuvants to enhance their immunogenicity. In this review, we focus on adjuvants that can enhance the immunogenicity of DNA encoded antigens and highlight the development of a novel cytolytic DNA platform encoding a truncated mouse perforin. The application of this innovative DNA technology has considerable potential in the development of effective vaccines.

## 1. Introduction 

Vaccines represent an effective strategy in the fight against infectious diseases and recent estimates suggest that vaccination prevents 2–3 million deaths every year [1]. The need for rapid and large scale vaccine production during epidemics against emerging pathogens is a major challenge in vaccine development [2], including effective vaccines for antigenically diverse and versatile pathogens that successfully subvert host immunity such as human immunodeficiency virus (HIV), hepatitis C virus (HCV), and malaria [3,4,5].

DNA vaccines can overcome some of these challenges, as it is relatively easy to produce large number of doses within a short period of time, and they are stable at ambient temperature and do not require cold chain transportation. They are also consistent between lots and have an excellent safety profile allowing for safety evaluations by regulatory authorities and distribution in a large scale [6,7]. Importantly, DNA vaccines can induce both humoral and cell-mediated responses in the vaccinated host [8,9,10]. Although they are safe and well tolerated, they are often poorly immunogenic and inefficacious in humans [11]. Therefore, recent studies on the advancements of DNA vaccines are focused on effective delivery and increasing the immunogenicity of the encoded antigen/s of interest [12,13].

Effective immunization with DNA vaccines requires efficient transfection of host cells which is highly dependent on the delivery route and use of devices. Conventional delivery routes to introduce the DNA vaccine include intramuscular, intradermal, subcutaneous and oral routes [12]. The preferred delivery route depends on the requirement to activate specific immune cells. The skin is rich in immune cells including local dendritic cells (DCs) and natural killer (NK) cells, and is therefore likely to be a more favorable site for vaccine delivery [14,15]. Attempts to improve DNA delivery have been made through other physical methods with the use of ‘gene guns’ or electroporation, which transiently permeabilizes the cell membrane to efficiently transfer the DNA resulting in increased vaccine uptake by skin and muscle cells [16]. Although these methods have shown to increase DNA uptake [17,18], they require optimization to achieve increased efficiency and acceptance for clinical use. An alternative approach to improve transfection efficiency includes formulation of DNA with liposomes or nanoparticles [19]. Liposomal delivery can be affected by pre-systemic (epithelial) and systemic barriers (enzymatic degradation, binding, and opsonization) [20]. Encapsulation of DNA with nanoparticles has been reported to increase DNA uptake or transfection efficiency [21,22]. Some of the challenges in the use of nanoparticles with DNA include encapsulation inefficiency, endocytosis by target cells and toxicity [23].

The use of genetic adjuvants is one approach to enhance the immunogenicity of the antigen and can be used to complement other strategies (e.g., DNA delivery) also designed to improve the immunogenicity of DNA vaccines. Upon immunization with a DNA vaccine, the target cells uptake DNA by endocytosis [24] and the transfected cells express the DNA-encoded protein antigen(s). When antigen-presenting cells (APCs) are directly transfected, the intracellular proteins are processed and immunogenic epitopes are then presented by MHC Class I molecules, which can directly stimulate naïve CD8^+^ T cells [12,25]. The protein immunogen released from transfected cells can be endocytosed and/or phagocytosed by other APCs and are presented by MHC class II molecules to activate naive CD4^+^ T cells [25,26]. If the proteins are expressed by stromal cells like keratinocytes, APCs can also indirectly capture secreted antigens and cross-present by MHC Class I molecules to further stimulate CD8^+^ T cells [27]. After DNA vaccination, CD8^+^ T cells specific to the vaccine antigen undergo expansion, acquire effector functions and differentiates into memory CD8^+^ T cells [28,29]. The memory cells differentiate into effector memory T cells upon re-exposure to the antigen [29,30]. The ability of a DNA vaccine to elicit T cell immunity is thus dependent on activating APCs to present antigen: MHC complexes to T cells [31] and adjuvants can serve as an important costimulatory factor to enhance this process.

In this brief review, based on our experience, we discuss the progress in the development of DNA vaccines, approaches to improve delivery and genetic adjuvants used to enhance immunogenicity. We focus on an innovative cytolytic DNA technology developed and patented in our laboratory.

## 2. DNA Vaccine Adjuvants

The immunogenicity of DNA vaccines is enhanced by CpG motifs present in the plasmid backbone, which can activate APC via toll like receptors (TLR9) [32,33,34]. Unmethylated CpG motifs have been reported to induce B cell proliferation and secretion of immunoglobulin in vitro and in vivo [33]. Activation of macrophages and DCs results in upregulation of antigen presentation and costimulatory molecules, and secretion of cytokines (IL-12 and IL-18) involved in helper T cells (Th1) response [34]. Thus, CpG motifs in DNA plasmids serve as a ‘natural adjuvant’ for DNA vaccines. Plasmid DNA can be designed to encode additional adjuvants with the antigen(s) of interest. Molecular adjuvants such as fusion proteins including heat shock protein 70 (HSP70), and vesicular stomatitis virus (VSVG) have been developed and used to enhance vaccine immunogenicity [35,36,37]. The gene encoding such proteins as adjuvants is either fused with the gene encoding the vaccine antigen to produce a fusion protein driven by a same promoter or as separate proteins driven by different promoters in the same or different plasmid. Co-encoding of genes creates a suitable cellular micro environment such as sustained antigen release and/or upregulation of cytokines, enhancing the immunogenicity of DNA vaccines [38]. 

A majority of the studies on experimental DNA vaccines with genetic adjuvants have been studied in animal models such as mice (Table 1), and very few of these have been clinically tested (Table 2). Limited published data on clinical trials pose difficulty to compare efficacy between adjuvants in animals and humans. 

### 2.1. Cytokines

Different cytokines, such as interleukins (IL-2, IL-6, IL-12), chemokines, granulocyte/macrophage colony-stimulating factor (GM-CSF), costimulatory molecules (CD40, CD80, and CD86), and signaling molecules (Interferon regulatory factor -3) have been used as genetic adjuvants with DNA vaccines [39,40,42,43,44,48,68]. Genes expressing IFN-γ IL-2, IL-12, IL-15, and IL-18 have been used to stimulate Th1 responses [44,45,70], and IL-4, IL-6, IL-10, IL-13, for Th2 stimulation [42,43,71,72,73]. The inclusion of genes encoding cytokines, like IL-2 or IL-12, as adjuvants for HIV-1 DNA vaccines is known to increase cell mediated immunity (CMI) [74,75]. However, a bicistronic HIV DNA encoding gp120 and IL-2 elicited weaker specific immune response than monocistronic HIV-1 gp120 DNA [76]. Combinations of genetic adjuvants like IL-2 and IL-15 with HIV-1 DNA vaccine have also been used but no synergistic effect on the level of total antibody to HIV-1 antigen was reported [77]. A phase I/IIa trial showed that coadministration of DNA vaccine encoding prostatic acid phosphatase (PAP) with GM-CSF elicited PAP-specific CD4^+^ and/or CD8^+^ T cell responses [68]. However, GM-CSF was administered as a recombinant protein.

### 2.2. Heat Shock Proteins

HSP70, a class of molecular chaperone, is known to induce maturation of DCs and activation of the Th1 pathway [78,79,80]. A fusion vaccine for multiple myeloma termed hDKK1-hHSP70 was shown to be effective in inhibiting the targeted tumor and increased survival of vaccinated mice by eliciting tumor-specific humoral and cellular immune responses [80]. However, a DNA vaccine encoding HPV16E7 fused with HSP70, targeting HPV16 and cervical intraepithelial neoplasia 2/3 failed to enhance significant T cell responses in a Phase I clinical trial [69]. A bicistronic DNA encoding HSP70 as a membrane bound or secreted protein has been used to improve the immunogenicity of a HIV Gag [60]. In this case, HSP70 expression was driven by a weaker SV40 promoter and HIV Gag by a stronger CMV promoter. Such a vaccine design enhanced Gag-specific T cell responses, providing greater protection in mice challenged with EcoHIV [60]. EcoHIV is a chimeric virus containing the envelope protein gp 80 of mouse leukemia virus rather than HIV gp 120 that can replicate in mouse leukocytes in vivo, thus representing a viable mouse challenge model for early assessment of HIV vaccines [81]. The proposed mechanism of HSP70 as an adjuvant is that TLR 2/4 on DCs interacts with secreted or bound HSP70, further attracting DCs to the site of antigen expression. This is followed by DC maturation, presentation of antigens by MHC molecules and secretion of cytokines and costimulatory molecules [82], thus enhancing T cell immune responses against the vaccine antigen.

### 2.3. Chicken Complement Inhibitor

A chimeric version of the oligomerization domain from the chicken complement inhibitor (C4bp) was used to produce an oligomeric form of vaccine antigens [35,83]. This protein, termed IMX313, forms a heptameric structure of the vaccine protein. This has been used to develop DNA vaccines for tuberculosis, malaria and HIV to enhance humoral and/or cellular responses [35,36,84]. Vaccination with a DNA vaccine encoding secreted HIV Tat (TPA-Tat IMX313) induced higher humoral and cellular responses and improved protection against EcoHIV challenge in mice [36]. A phase I clinical trial of tuberculosis vaccine MVA85A-IMX313 evaluated the vaccine to be safe and immunogenic, but cellular (Ag85A-specific IFN- Ƴ) and humoral (MVA-specific IgG) responses were not significant [85]. Thus, the ability of such an adjuvant to enhance immunogenicity of DNA vaccines in humans is still debated.

### 2.4. Viral Fusion Protein

VSV G is a type III viral fusion envelope protein, which mediates fusion of the virus envelope and the host cell membrane [86]. The use of fusogenic membrane glycoprotein (FMG) gene from VSV in a DNA vaccine encoding the H7 protein of human papillomavirus type 16 was shown to enhance CD8^+^ T cell responses and effectively control growth of tumors [87]. The VSV G protein was also shown to induce a T-response at low doses or a T-independent response at higher doses [88]. FMG fuses cells into large multinucleated syncytia, which are then killed by a nonapoptotic mechanism [89]. Syncytiosomes are released from the membrane and present antigens efficiently to DCs [90]. FMGs can thus act as an adjuvant for any antigen expressed by a DNA vaccine. Vaccination of mice with the hepatitis C virus (HCV) nonstructural protein 3 (NS3) together with VSVG resulted in increased frequency of IFN- γ, but not TNF-α- or IL-2-producing CD3+ CD44+ CD8+ effector memory T (T_EM_) cells [57]. This DNA vaccine was constructed in a bicistronic vector with VSVG co-encoded in the same plasmid with HCV NS3. VSVG expression was driven by a weaker SV40 promoter and NS3 by a stronger CMV promoter [57]. However, others have also reported an increase in the specific CMI when the immunogen and VSVG were expressed from different plasmids [37,87].

### 2.5. Cytolytic Protein

Perforin (PRF) is a pore forming protein released by immune cells including NK cells and cytotoxic T lymphocytes (CTL) [91]. The 67-kilodalton PRF protein oligomerizes to form pores that serve as a channel to release granzyme into the cytosol of target cells [92]. Suicide genes inducing apoptosis of target cells have been used for cancer therapies, and the role of apoptotic cell death in vaccination, whether immune-stimulatory or immune-suppressive, is debated [93]. Recently, a novel DNA technology has been developed––termed cytolytic DNA technology—in which a truncated mouse PRF is incorporated in a bicistronic DNA vector to act as a vaccine adjuvant [55,56,57,94].

## 3. Cytolytic DNA Vaccines

**Figure 1:**
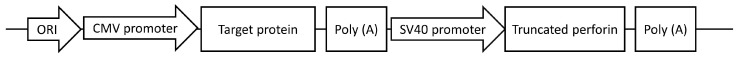
Schematic diagram of cytolytic bicistronic DNA plasmid (rDNA-PRF) with two different promoters and encoding protein of interest (immunogen) and truncated perforin.

Cytolytic DNA vaccines are based on a bicistronic DNA plasmid constructed on a pVax backbone (Invitrogen) with a Cytomegalovirus (CMV) promoter and a Simian Virus (SV40) promoter [55]. The gene encoding the protein of interest as an immunogen is inserted downstream of the CMV promoter and the gene encoding a truncated version of mouse PRF downstream of the SV40 promoter (Figure 1). The SV40 is a weaker promoter compared to CMV and has been shown to result in 10-fold lower protein expression in transfected HEK293T cells in vitro [55,95].

The PRF gene was modified to express a truncated version of PRF (~60KDa) lacking the final 12 amino acid residues of the C terminus (unstructured region of PRF) [56,57,94] in order for PRF to become cytolytic [96]. The final 12 C-terminal amino acids are required to export PRF protein from the endoplasmic reticulum (ER) to the Golgi, from where glycosylated PRF is then transported to secretory granules [96]. Removal of the C-terminal abrogates the export of PRF from the ER and its subsequent accumulation in the ER is cytotoxic to the host cell [96,97].

### 3.1. Mechanism

Different recombinant cytolytic DNA-PRF vaccines (rDNA-PRF) have been shown to elicit immune responses higher than those elicited by canonical DNA vaccines (without PRF) and the mechanism underlying this has been established [94]. A previous study showed that coexpression of HCV NS3 and PRF elicited nonapoptotic cell death in transfected cells, whilst immunization with NS3-PRF DNA vaccine increased NS3-specific T cell mediated responses as evidenced by increased NS3-specific IFN-γ responses in an ELISpot assay and increased numbers of polyfunctional CD8^+^ T_EM_ cells that simultaneously secreted IFN-γ, IL-2, and TNF-α [56]. Cytolytic DNA platform where the expression of immunogen is driven by a stronger promoter allows for sufficient antigen expression and accumulation within the target cells followed by nonapoptotic cell death due to lesser expression of PRF driven by a weaker SV40 promoter; thus, balancing the level of antigen expression with the timing of cell death [94]. 

Necrosis is considered as the mechanism of cell death by PRF as evidenced by release of lactate dehydrogenase (LDH) and low caspase activity [55,56,94]. LDH release occurs after the rupture of cell membrane during secondary necrosis [98,99]. In contrast, LDH was not released by cells treated with doxorubicin (a proapoptotic drug) or cells transfected with NS3 wild type PRF or NS3 12del483A PRF (mutant and nontoxic PRF) [94]. Expression of PRF from a cytolytic DNA, e.g., NS3 PRF vaccine, thus results in necrotic cell death mediated by receptor-interacting protein-1 kinase activity, as evidenced by detection of uncleaved cytokeratin 18 in Huh-7 cells [56]. Necrosis releases damage associated molecular patterns (DAMPs) which in turn activate DCs to migrate to the site of vaccination [100,101]. 

**Figure 2:**
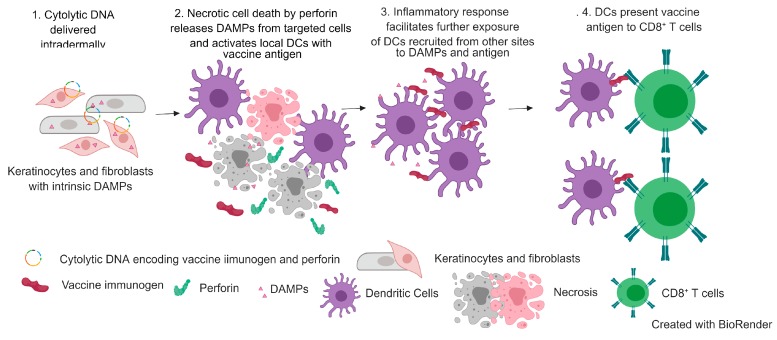
Mechanism of rDNA-PRF immunogenicity.

When purified DCs including the CD8α^+^ subset from naïve C57BL/6 mice were exposed to HEK293T cells (transfected with Ovalbumin-PRF), it resulted in upregulation of costimulatory molecules (CD80/CD86), indicating maturation of the immune cells with the cytolytic DNA [94]. A significant increase in CD11c^+^ DCs and cross-presenting CD8a^+^ DCs, and upregulation of CD80 has been reported in mice vaccinated with a cytolytic DNA HIV 1 Gag PRF compared to a canonical DNA vaccine [55]. Local and migrated DCs at the site of inflammation can take up antigens by endocytosis and are also exposed to DAMPs. Activated and matured DCs can then prime naïve CD8^+^ T cells (Figure 2). Antigen cross-presentation by DCs to CD8^+^ T cells has been shown to increase the number of proliferating CD8^+^ T cells by ~2-fold with cytolytic DNA compared to the noncytolytic PRF DNA [94]. Thus, a cytolytic DNA vaccine has an inbuilt adjuvant to enhance the immunogenicity of the vaccine immunogen. Whereas, the immunogenicity of canonical DNA vaccines mostly depends on direct transfection of DCs and to a lesser extent cross-presentation of antigens shed from transfected cells and/or derived from transfected cells that have undergone spontaneous cell death [94].

Several studies have established that DCs exposed to necrotic or lytic cells expressing antigens mature and cross-present more efficiently than DCs exposed to antigens derived from a cellular milieu that comprise of apoptotic cells [102,103,104,105,106]. Comparative studies evaluating the ability of proapoptotic (e.g., rotavirus nonstructural protein 4 (NSP4) and diphtheria toxin subunit A (DTa)) and necrotic proteins (e.g., truncated PRF) to enhance the immunogenicity of DNA when encoded in plasmid DNA vaccines showed that truncated PRF is the most effective for this purpose [55,56,57]. However, a caveat is that vaccine-encoded antigens need to accumulate significantly inside the cell before necrosis occurs following expression of truncated PRF in order to activate DCs to cross-present vaccine-encoded antigens [55,94].

### 3.2. Cytolytic HIV and HCV DNA Vaccines

rDNA-PRF technology has been used in the development of HIV and HCV DNA vaccines [55,57,107]. Direct comparison of the effects of the cytolytic PRF and the apoptotic protein DTa on the immunogenicity of the HIV-1 Gag protein showed that PRF activated DCs more efficiently, as evidenced by the increase in frequency of cross-presenting DCs and upregulation of activation marker (CD80) [55]. In both DNA vaccines, PRF and DTa were driven by SV40 promoter. Immunization of mice with a DNA vaccine encoding proapoptotic DTa as an adjuvant in a HIV Gag DTa vaccine resulted in decreased DC activation, suggesting that DTa-induced apoptosis attenuated immune response [55]. Furthermore, improved protection in the mouse EcoHIV challenge model was achieved with rDNA-PRF encoding HIV Gag compared to protection levels in mice vaccinated with a canonical Gag DNA vaccine [57]. A rDNA-PRF vaccine encoding the HCV NS3 protein coexpressed with PRF was shown to increase NS3-specific CMI in mice and pigs, compared to NS3 coexpression with a proapoptotic protein, the rotavirus NSP4 protein [56]. NSP4 is an enterotoxin that elicits a proapoptotic effect by disrupting the mitochondrial membrane and activating caspase-3, -8, and -9 [108,109]. This study showed that PRF coexpression induced cell death by necrosis, and thus enhanced NS3-specific immune responses, whereas, proapoptotic NSP4 reduced NS3-specific response [56]. Importantly, HCV NS3 PRF was more immunogenic than the canonical NS3 vaccine in pigs, demonstrating the translational potential of the cytolytic DNA vaccines in human clinical trials [56]. Likewise, we have shown that a multi-antigenic HCV DNA vaccine encoding genotype 3a proteins NS3, NS4A, NS4B, and NS5B coexpressed with PRF induced robust CMI against the range of HCV NS proteins, compared to coexpression with VSVG [57]. We also showed that multi-antigenic and multigenotypic (HCV genotype 1 and 3a) DNA cocktail vaccines encoding PRF can significantly increase the magnitude and breadth of CMI responses to NS3 and NS5B against both genotypes compared to those elicited by a single-genotype vaccine [107].

## 4. Conclusions

DNA vaccines are still a promising option in the development of novel vaccination strategies. Although they have many advantages, the ability to induce effective immune responses in humans required for protection has been challenging. These challenges include ineffective delivery and poor uptake of DNA. Consequently, a recent focus has been in the development of delivery methods and/or inclusion of genetic adjuvants. Such genetic adjuvants are generally coexpressed with the antigen of interest or delivered through different plasmids. In the quest to develop and identify effective genetic adjuvants, a range of adjuvants was tested (HSP70, VSVG, IMX313, DTa, and PRF) and a novel and promising cytolytic DNA vaccine strategy has been developed. This cytolytic DNA vaccine is unique as it is based on a bicistronic plasmid with the ability to coexpress antigen and PRF in a balanced mechanism causing necrosis of vaccine-transduced cells, followed by increased activation of immune cells and cross presentation of vaccine immunogen. Cytolytic DNA vaccines encoding nonstructural proteins of HCV have been tested to enhance immunogenicity of vaccine antigen in mice [57,107] and in a large preclinical animal model, the pig [56]. Likewise, increased immunogenicity and improved protection against EcoHIV challenge in mice with HIV Gag PRF [60] demonstrate the effectiveness of cytolytic DNA vaccines.

Adjuvants that provide effective costimulation for immune responses with specific immunogens may not have a similar effect with other immunogens, and therefore these need to be tested for their efficacy. Use of a genetic adjuvant such as PRF produces a suitable microenvironment for multiple/different immunogens and thus improves the delivery, immunogenicity and effectiveness of DNA vaccines. This strategy has considerable potential in the development of DNA-based vaccines against a range of infectious agents.

## Figures and Tables

**Table 1:** Molecular adjuvants and immunogenicity of DNA vaccines in animals.

Adjuvants	Antigens	Delivery	Host	Responses	Ref.
**Costimulatory molecules**					
CD80, CD86	HIV-1 (Env, Gag, Pol)	DC, IM	Mouse Chimpanzee	+CMI	[39]
CD40 LT	beta-gal	DC, SC	Mouse	+Ab, +CMI	[40]
ICAM-1	HIV-1 (Env)	DC, IM	Mouse	+CMI	[41]
**Cytokines**					
IL-2, IFN-γ	HIV-1 (Env, Gag, Pol)	DC, IM	Mouse	+Ab, +CMI	[42]
IL-6	Influenza (HA)	DC, GG	Mouse	+Ab	[43]
IL-2,12, IFN-γ	HBV	DC, IM	Mouse	+CMI	[44]
TNF-α, IL-15	HIV (Env, Gag, Pol)	DC, IM	Mouse	+CMI	[45]
**Toll like receptor adaptor/signaling molecules**					
TRIF	Influenza (HA), tumor E7	BC, IM/EP	Mouse	+CMI	[46]
MyD88	Influenza (HA), tumor E7	BC, IM/EP	Mouse	+Ab	[46]
FliC	Influenza A (Np)	DC, ID	Mouse	+Ab, +CMI	[47]
IRF 1,3, 7	Influenza virus (HA, Np)	DC/BC, IM	Mouse	+Ab, +CMI	[48]
TBK-1	P. f (SE36)	DC, IM	Mouse	+Ab, +CMI	[49]
HMGB1	HIV-1 (Gag, Env)	DC, IM/EP	Mouse	+Ab, +CMI	[50]
DAI	Survivin	DC, ID	Mouse	+CMI	[51]
chMDA5	Influenza (HA)	DC, IM	Chicken	+Ab	[52]
Ii	P. f (ME)	FC, IM	Mouse	+CMI	[53]
**Toxins/Viral proteins**					
FrC	Sc-fv	FC, IM	Mouse	+Ab	[54]
DTa	HIV (Gag)	BC, ID	Mouse	−CMI	[55]
NSP4	HCV NS3	BC, ID	Mouse	+/−CMI	[56]
VSVG	HIV (Gag) NS3	DC, ID BC, ID	Mouse Mouse	+CMI +/−CMI	[37] [57]
**Heat shock proteins**					
Calreticulin	mucin 1 HPV-16 E7	DC FC, GG	Mouse Mouse	+CMI +CMI	[58] [59]
HSP70	HIV (Gag)	BC, ID	Mouse	+CMI	[60]
**Complement inhibitor**					
IMX313	HIV (Tat)	FC, ID	Mouse	+Ab, +CMI	[36]
**Cytolytic protein**					
PRF	HIV (Gag) HCV (NS3) HCV (NS345B)	BC, ID BC, ID BC, ID	Mouse Mouse, Pig Mouse	+CMI +CMI +CMI	[55] [26] [57]

**Adjuvants:** LT: ligand/trimer, IL: Interleukin, TNF: Tumor necrosis factor, TRIF: Toll-interleukin-1 receptor domain-containing adaptor-inducing beta interferon, MyD88: myeloid differentiation primary response, FliC: phase-1 flagellin, IRF: Interferon regulatory factor, TBK-1: TANK-binding kinase 1, HMGB1: High-mobility group box 1 protein, DAI: DNA-dependent activator of interferon (IFN) regulatory factors, chMDA5: melanoma differentiation-associated gene 5 product, FrC: Fragment C of tetanus toxin, DTa: Diphtheria toxin subunit A, NSP4: Nonstructural protein 4, li: MHC class II invariant chain, HSP: Heat shock protein, VSVG: Vesicular stomatitis virus, PRF: Perforin; **Antigens:** HIV: Human immunodeficiency virus, Env: Envelope, GAG: Group antigens, Pol: Reverse transcriptase, beta-gal: beta galactosidase, FMDV: Foot and Mouth Disease Virus, VP1: Virus protein 1, HBV: Hepatitis B virus, HA: Haemagglutinin, Sc-fv: Single chain fragment variable, Np: Nucleoprotein, P.f: *Plasmodium falciparum*, SE36: serine repeat antigen 36, HCV: Hepatitis C virus, NS3: Nonstructural protein 3, ME: Multiepitope string fused to the native *P. falciparum* T9/96 strain,NS345B: Nonstructural proteins 3, 4, 5B; **Delivery:** DC: Different constructs, BC: Bicistronic construct, FC: Fusion protein/single construct, IM: Intra muscular, SC: Subcutaneous, GG: Gene gun, EP: Electroporation, ID: Intradermal; Responses: +: Increase, −: Decrease, +/−: No significant change, CMI: T cell responses, Ab: Humoral responses; **Ref.:** References.

**Table 2:** Molecular adjuvants tested with DNA vaccines in humans.

Adjuvants	Antigens	Delivery	Responses	Trial Phase	Ref.
IL-12, IL-15	HIV-1 (Gag)	DC, IM	+/−Ab, +/−CMI	I	[61]
GM-CSF, IL-2	Her2	RP, IM	+Ab, +CMI	I	[62]
GM-CSF	CEA	RP, ID	+Ab, +CMI	I	[63]
IL-2/Ig	HIV-1 Gag/Pol/Nef/Env	BC, IM	+Ab, +CMI	I	[64]
IL-12	HIV (MAG-Gag, Pol, Env, Nef, Tat, Vif)	DC, IM/EP	−Ab, +CMI	I	[65,66]
IL-12	HIV-1 (Env, Gag, Pol)	DC, IM/EP	+CMI	I	[67]
GM-CSF	PAP	RPID	−Ab, +CMI	I/IIa	[68]
HSP70	HPV16 (E7)	FC, IM	−Ab, +/−CMI	I	[69]

**Adjuvants:** IL: Interleukin, GM-CSF: Granulocyte/macrophage colony-stimulating factor; **Antigens:** HIV: Human immunodeficiency virus, Gag: Group antigens, Her2: Human epidermal growth factor receptor 2, CEA: Human carcinoembryonic antigen, MAG: Multi antigen, Env: Envelope, Pol: Reverse transcriptase, Nef: N-terminally myristoylated protein, Tat: Transactivator of transcription, Vif: viral infectivity factor, PAP: Prostatic Acid Phosphatase, HSP: Heat shock protein, HPV: Human Papilloma Virus; **Delivery:** DC: Different constructs, BC: Bicistronic construct, FC: Fusion protein/single construct, RP: Adjuvant as recombinant protein, IM: Intramuscular, ID: Intradermal; Responses: +: Increase, −: Decrease, +/−: No significant change, Ab: Humoral responses, CMI: T cell responses; **Ref.:** References.
